# The Research Hotspots and Future Trends of Adaptive Learning in the Age of Artificial Intelligence: A Bibliometric Analysis From 2014 to 2024

**DOI:** 10.1155/jonm/6689213

**Published:** 2025-08-18

**Authors:** Nanxi Kang, Lu Liu, Shan Zhang

**Affiliations:** Capital Medical University School of Nursing, Beijing, China

**Keywords:** adaptive learning, artificial intelligence, bibliometric analysis, education

## Abstract

**Aims:** To examine the research status and developmental trend within the adaptive learning.

**Background:** Adaptive learning provides personalized learning paths based on the unique needs of each learner. However, a comprehensive bibliometric analysis of this field remains limited.

**Methods:** A bibliometric analysis was conducted. Our search within the Web of Science database targeted articles on adaptive learning published from January 1, 2014, to November 16, 2024. The dataset encompassed publication counts, participating countries, institutions, authors, cited journals, references, and keywords, with CiteSpace facilitating the bibliometric analysis.

**Results:** The review encompassed 561 articles by 288 authors across 240 institutions in 68 countries. These publications showed an upward trend over the decade, with the United States of America leading with 214 articles (38.15%). The University of Toronto topped institutional contributions with 16 articles (2.85%). *Computers and Education* emerged as the most cited journal in adaptive learning, with 244 articles. Timeline analysis and burst detection identified key research hotspots, including the theoretical and technological underpinnings of adaptive learning, its educational applications, and its efficacy. Emerging trends suggest a shift toward intelligent optimization and outcome-focused adaptive learning, as well as its integration with higher education.

**Conclusions:** The study provides a comprehensive view of adaptive learning research from the past decade, offering insights and indicating future research directions within the field.

**Implications for Nursing Management:** Nursing administrators leverage adaptive learning mechanisms to intelligently identify knowledge gaps in nursing practice, develop personalized training programs and simulation exercises, and optimize nursing management models.

## 1. Introduction

Since the 1950s, the volume of medical knowledge has seen a dramatic increase, from doubling every half-century to doubling every 3.5 years by the early 21st century. Today, the knowledge base doubles approximately every 73 days [1]. In the era of information explosion, an increasing number of educators are integrating artificial intelligence (AI) technologies into the learning process of students, such as virtual reality (VR), augmented reality (AR), virtual hospitals, and remote teaching methods [2, 3]. The explosive growth of online education has transformed traditional teaching models, making it a reality for students to learn anytime, anywhere, and enhancing their interest and self-learning abilities [4, 5]. Additionally, the rapid expansion of medical knowledge and the increasing complexity of healthcare demands necessitate innovative approaches to nursing education and workforce management [6]. Traditional training models often struggle to address the diverse learning needs of nursing professionals, particularly in fast-paced clinical environments [7].

However, the differences in each learner's knowledge base, needs, experiences, and interests indicate that only by allowing everyone to learn in the most suitable way for them can educational goals and personal development be better achieved [8]. Adaptive learning, powered by AI, presents a transformative opportunity for nursing management by enabling personalized, data-driven professional development. AI-driven adaptive learning systems can analyze nurses' learning behaviors, clinical performance, and knowledge gaps, dynamically adjusting training content to optimize competency acquisition [9, 10]. Furthermore, these technologies facilitate intelligent workforce allocation by predicting workload fluctuations and optimizing shift scheduling, ensuring both care quality and staff well-being [10]. By integrating adaptive learning into nursing education and management, healthcare institutions can enhance training efficiency, reduce skill disparities, and foster a more resilient nursing workforce [9]. As the volume of educational data expands exponentially, the ability to harness these data to inform teaching strategies and enhance learning outcomes becomes paramount [11]. Adaptive learning not only improves learning efficiency, ensuring that learners master knowledge at an appropriate difficulty level, but also enhances motivation, making the learning process more interesting and effective [12, 13].

Despite the burgeoning interest in adaptive learning, a comprehensive bibliometric analysis of this field remains limited. With the proliferation of online learning platforms and the rise of AI in education, adaptive learning has become a cornerstone in the quest for efficient and effective pedagogical solutions [14]. To pinpoint key areas for research and development, a systematic and comprehensive approach is essential. Bibliometric analysis, a methodological framework that employs quantitative techniques to assess scholarly literature, offers a powerful tool for mapping the hotspots of research domains [15]. Our study aims to analyze the Web of Science (WoS) data and utilize CiteSpace software to conduct a bibliometric analysis of the adaptive learning literature [16]. By reporting publications, authors, countries, institutions, cited journals, and keywords, the study seeks to outline the research hotspots and anticipate future trends of adaptive learning.

## 2. Materials and Methods

### 2.1. Data Source and Search Strategy

The WoS Core Collection was chosen for its comprehensive coverage of high-impact journals and multidisciplinary relevance to adaptive learning research. The query combined synonyms of adaptive learning: “TS = (“adaptive learning” or “learning adaptive” or “adaptive learning technology” or “learning adaptive technology” or “adaptive learning platform” or “adaptive educational technology” or “personalized intelligent learning system” or “intelligent tutoring system” or “intelligent e-learning system”).

### 2.2. Inclusion and Exclusion Criteria for Literature

The search was limited to documents categorized under “education scientific disciplines” and “education educational research” within the WoS database. We included peer-reviewed articles/reviews in English from January 1, 2014, to November 16, 2024. Conference abstracts, editorials, and nonrelated studies were excluded. After the initial search, two researchers independently reviewed the abstracts, and when necessary, the full texts of the literature on adaptive learning were examined to eliminate off-topic studies. Any discrepancies in the selection process were resolved through discussion among the authors and by seeking expert consultation to achieve consensus. Ultimately, 561 articles met the inclusion criteria.

### 2.3. Data Analysis

Both descriptive statistics and bibliometric analyses were performed using CiteSpace Version 6.3.R1 [16]. This software enabled the quantification of annual publication outputs in adaptive learning research and visualized their geographical distribution. The time slicing parameter was configured with annual intervals (2014–2024) to track temporal trends. Node types included countries, institutions, authors, cited authors, cited journals, and references, with the top 50 nodes per slice set as the threshold. Cosine similarity was selected to measure network connectivity strength. High-frequency keywords were extracted, with the top 30 identified using the minimum spanning tree method and a pruning slice network to reduce complexity while retaining key connections. Keyword clustering was conducted using the log-likelihood ratio test, where modularity (*Q*) > 0.3 confirmed significant cluster structure and silhouette (*S*) > 0.5 indicated high within-cluster homogeneity [17]. For burst keyword detection, parameters included gamma (*γ* = 1.0), a sensitivity level of 2.0, and a minimum duration of 1 year to identify emerging trends. The E-value, reflecting interdisciplinary diversity based on knowledge graph connections, was analyzed, and higher values denote greater thematic diversity. Additionally, the density value, calculated as the ratio of actual connections to maximum possible connections, was assessed, with values closer to 1 indicating stronger internode connectivity.

### 2.4. Ethical Consideration

This study is based on secondary data analysis using publicly available bibliometric databases, and it does not involve direct interaction with human participants.

## 3. Results

### 3.1. Publication Outputs of Adaptive Learning Research

**Figure 1:**
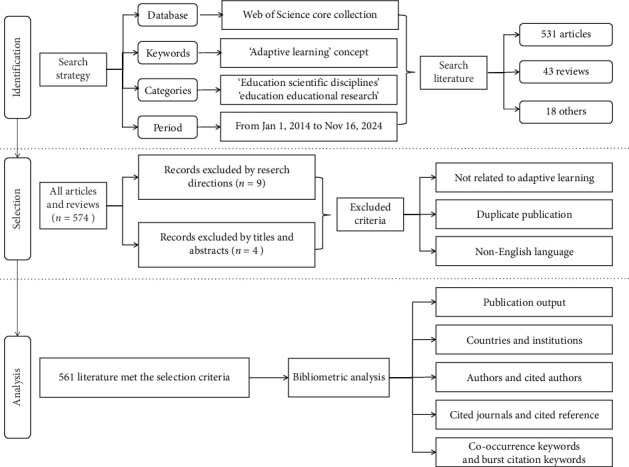
Flowchart of the literature-screening process.

A search of the WoS Core Collection database identified a total of 592 documents. After applying the literature-screening criteria, 561 publications were deemed suitable for the final analysis, as shown in Figure 1.

**Figure 2:**
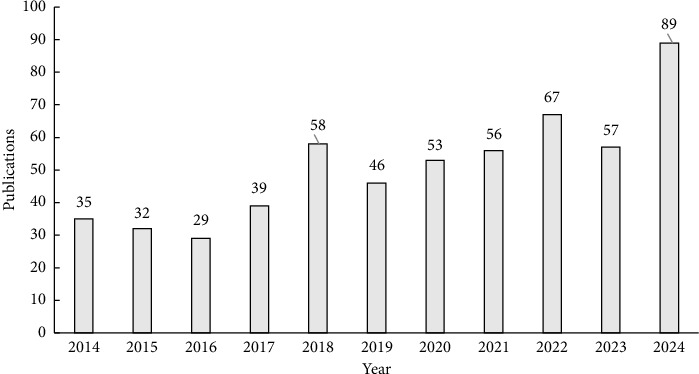
Publication output related to adaptive learning research by year from 2014 to 2024.

The trend analysis of adaptive learning publications reveals a fluctuating upward trend. Post-2020, the annual output has steadily exceeded 50 publications, culminating in a peak of 89 articles in 2024 (as shown in Figure 2).

From 2022 to 2024, the publication count totaled 213, constituting 37.97% of all articles reviewed. This significant rise in academic engagement reflects an escalating emphasis on adaptive learning research.

### 3.2. Publication Countries in Adaptive Learning Research

Sixty-eight countries contributed to the publications on adaptive learning. The connectivity among these nations, measured by the connectivity index, totaled 157, with a density of 0.0689, suggesting a moderate level of collaborative engagement.  Table 1 outlines the top 10 countries in terms of contribution, with the United States at the forefront, publishing 214 articles, which represents 38.15% of the overall count. China secured the second position with 58 articles (10.34%), succeeded by Canada with 45 articles (8.02%), and the Netherlands with 32 articles (5.70%).

### 3.3. Publication Institutions in Adaptive Learning Research

A total of 240 institutions participated in adaptive learning research publications, resulting in 241 collaborative efforts among them and a density of 0.0084.  Table 2 shows the leading 10 institutions based on their publication volume in this field. The University of Toronto, Canada, led the ranking with 16 papers, constituting 2.85% of the overall total. It was succeeded by New York University, USA, with 13 papers (2.32%), and Purdue University and the University of Michigan, both from the United States if America, each contributing 12 papers (2.14%).

### 3.4. Core Authors in Adaptive Learning Research

A total of 288 authors have participated in the field of adaptive learning research, forging 311 collaborative ties and achieving a density of 0.0075 among their network.  Table 3 highlights the top 10 authors based on their publication frequency. At the top is Mylopoulos, Maria from the University of Toronto, Canada, who has authored 13 papers in adaptive learning research, constituting 2.32% of the total contributions.

### 3.5. Cited Authors in Adaptive Learning Research

Network analysis identified 242 cited authors, characterized by an E-value of 1238 and a density of 0.0425.  Table 4 ranks the most-cited authors, with the top three being Brusilovsky P, who has 42 publications (7.49%) and is affiliated with the University of Pittsburgh, USA; Zimmerman BJ, contributing 41 publications (7.31%) from Millersville University, USA; and Cohen J, with 40 publications (7.13%), associated with Princeton University, USA.

### 3.6. Cited Journals in Adaptive Learning Research

Two hundred and six cited journals have featured research on adaptive learning within the study's timeframe, highlighting their substantial influence in this academic domain.  Table 5 lists the top 10 most-cited journals in adaptive learning research. Leading the list is *Computers and Education*, which has published a significant number of 244 articles (43.49%) with an impact factor of 8.9. *Computers in Human Behavior* follows with 157 articles (27.99%), and *Medical Education* contributes 135 articles (24.06%), both of which have significantly enriched the body of literature on adaptive learning.

### 3.7. Cited Reference in Adaptive Learning Research

Adaptive learning research has been significantly shaped by 216 key references that provide researchers with a rapid understanding of the research landscape, central themes, and evolutionary trends within the discipline.  Table 6 highlights the top 10 most-cited references, reflecting their influential role in the body of the adaptive learning literature as indicated by their frequency of citation.

### 3.8. Keyword Analysis

**Figure 3:**
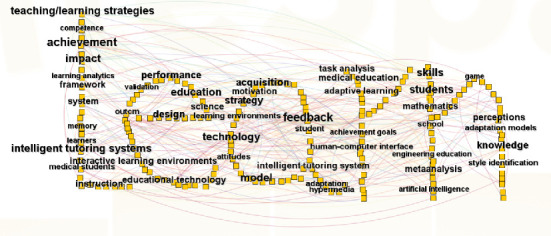
Keyword co-occurrence network.

The study identified 36 keywords that occurred at least 10 times. These 291 keywords were visualized in a graphical format, as depicted in Figure 3. Central nodes such as intelligent tutoring systems (80 counts) and performance (64 counts) reflect dominant research themes.

Table 7 highlights the 20 most prominent keywords reflective of the discourse on adaptive learning within the scholarly literature. High-betweenness centrality terms (e.g., impact/feedback/achievement, centrality = 0.20) act as bridges connecting subfields.

**Figure 4:**
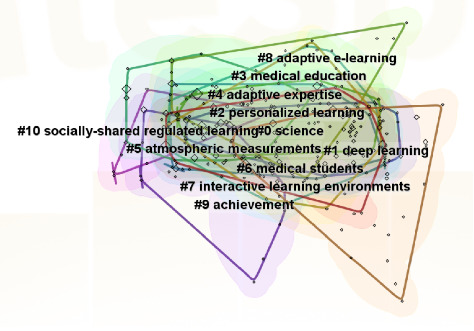
Keywords clustering graph of adaptive learning research.

The keyword clustering analysis yielded an *S*-value of 0.8213 and a *Q*-value of 0.5362, indicating the validity and relevance of the clusters to the research trends in adaptive learning.  Figure 4 illustrates distinct color-coded clusters, offering a clear visual distinction among different groups, with keywords categorized into 11 distinct thematic groups. For example, Cluster #0 science focuses on knowledge analytics and personalized feedback, Cluster #1 deep learning highlights AI-driven methods, and Cluster #3 medical education ties adaptive learning to clinical training. Overlaps between #2 (personalized learning) and #8 (adaptive E-learning) suggest synergies in AI-aided customization.

**Figure 5:**
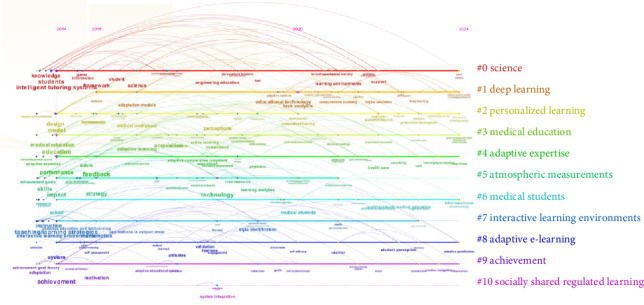
Keywords co-occurrence timeline graph of adaptive learning research.

Figure 5 shows 11 clusters within the adaptive learning research, with each cluster demarcated by its keywords. Cluster 0, dedicated to science, encompasses keywords such as “knowledge, information, competencies, personalized feedback, analytics, and technology-enhanced learning.” Cluster 1, centered on deep learning, includes keywords such as “patterns, adaptive systems, computer-aided instruction, machine learning, algorithm, and hidden Markov models.” Cluster 2, concerning personalized learning, includes keywords such as “design, AI, cognitive load theory, flipped classroom, challenge, and knowledge graphs.” Cluster 3, which focuses on medical education, highlights “gross anatomy education, recommender systems, decision-making, self-directed learning, adaptive learning, and competence.” Cluster 4, on adaptive expertise, comprises keywords such as “performance, feedback, outcome, adaptive comparative judgment, formative assessment, care, and physicians.” Cluster 5, related to atmospheric measurements, is characterized by “student-centered learning, learning analytics, big data, multimedia learning, technology, skills, strategies, and impact.” Cluster 6, on medical students, contains keywords such as “anxiety, cognitive load, undergraduate medical education, self-determination theory, intrinsic motivation, school, and public health.” Cluster 7, related to interactive learning environments, is marked by “teaching/learning strategies, humancomputer interface, distance education and telelearning, and game-based learning.” Cluster 8, on adaptive e-learning, is identified by “adaptive gamification, online learning, student perception, retention, academic achievement, engagement, resilience, and burnout.” Cluster 9, on achievement, contains keywords such as “adaptation, satisfaction, self-assessment, emotion recognition, learning strategy, stress, motivation, and academic performance.” Cluster 10, related to socially shared regulated learning, is characterized by “worked examples, system integration, and questions.” Early phase (2014–2018) dominated by foundational theories (science and task analysis), and recent phase (2019–2024) shift toward AI applications and higher education integration.

The thematic analysis of adaptive learning research, facilitated by keywords and keyword clustering, has pinpointed three research focal points. The first category, related to theoretical basis and technology of adaptive learning, includes keywords such as model, design, task analysis, and science. The keyword clustering includes #0 science, #1 deep learning, #4 adaptive expertise, and #8 adaptive e-learning. The second category, focusing on the application of adaptive learning in education, includes keywords such as system, intelligent tutoring systems, and teaching/learning strategies. The keyword clustering includes #2 personalized learning, #3 medical education, #6 medical students, and #7 interactive learning environments. The third category, which evaluates the effect of adaptive learning, includes keywords such as performance, impact, feedback, motivation, and achievement. The keyword clustering includes #5 atmospheric measurements, #9 achievement, and #10 socially shared regulated learning.

**Figure 6:**
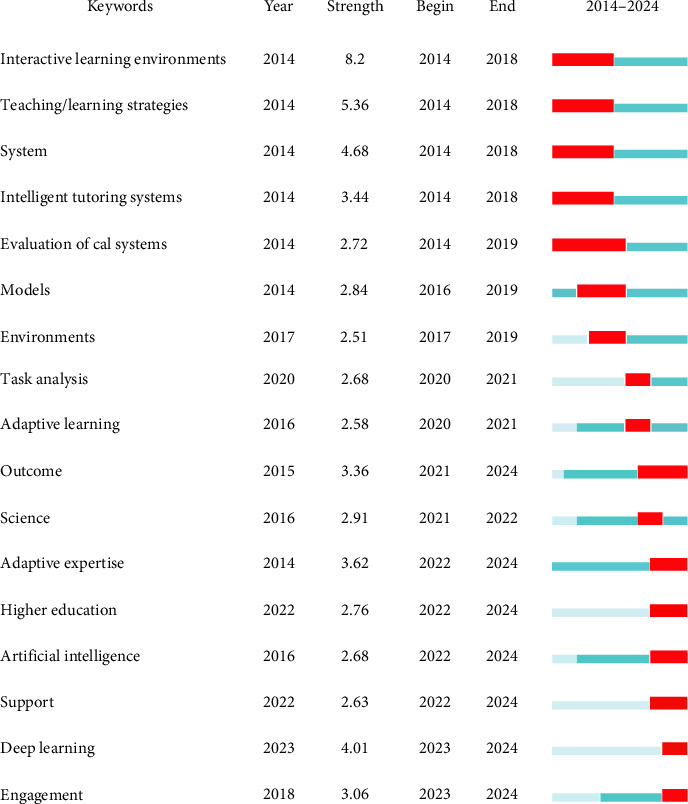
Top 17 keywords with strong citation bursts between 2014 and 2024.

Burst keywords, indicative of burgeoning research interests, highlight the latest advancements and trends in adaptive learning research.  Figure 6 displays the top 17 burst keywords from 2014 to 2024, with the red line's intensity signifying the heightened interest periods for each keyword. “Interactive learning environments” topped the list with the highest intensity score of 8.2, succeeded by “teaching/learning strategies” at 5.36 and “system” at 4.68. The keyword “AI” (burst strength = 2.68, 2022–2024) highlights AI's expanding role in adaptive learning, while “adaptive expertise” (burst strength = 3.62) reflects its critical implications for nursing management, particularly in enhancing skill retention, facilitating knowledge transfer, and fostering continuous learning among healthcare professionals. Additionally, burst keywords in recent years included “outcomes,” “adaptive expertise,” “higher education,” “artificial intelligence,” “support,” “deep learning,” and “engagement.” These keywords illuminate the current research trajectories and may evolve into pivotal topics for future investigative efforts.

## 4. Discussion

Our bibliometric review of adaptive learning publications from 2014 to 2024 charts an increasing research interest, with a fluctuatingly increasing trend in publication output. The United States leads in research output, accounting for 38.15% of the total, highlighting its significant role in this domain. The top 10 productive institutions are predominantly in technologically advanced nations such as Canada, the United States of America, and China, underscoring their influence on adaptive learning. The collaborative nature of the field is evident, with 288 authors from 240 institutions in 68 countries contributing to 561 papers, underscoring the need for intensified global and institutional collaboration. Among the journals, *Computers and Education* stands out as the most frequently cited, focusing on educational technology's latest research, innovations, and theoretical advancements.

AI can provide technical support for adaptive learning, and algorithms can analyze students' learning data, predict learning needs, and automatically adjust the content and difficulty of learning materials [18]. Additionally, adaptive learning technologies can analyze nurses' clinical performance data to build dynamic competency models, identifying gaps in knowledge or skills [19]. This aligns with Cluster #4 adaptive expertise, enabling personalized training paths. Theoretical basis and technology of adaptive learning is one of the research hotspots in the field of adaptive learning. For instance, with the surge in online learning resources, personalized resource recommendation has become particularly important. Ontology, as a knowledge modeling tool, helps to provide more relevant learning material recommendations, addressing the issue of information overload.

The use of ontologies can standardize nursing knowledge domains, e.g., infection control, patient safety, and automate recommendations for training content, reducing administrative burdens. The research indicates that ontology offers advantages in enhancing the reusability, inferencing capabilities, and support for inferential mechanisms of recommendation systems [20]. Rijgersberg-Peters et al. [21] have constructed an adaptive learning technology model that correlates individual knowledge elements, such as skills, with educational tasks and learner knowledge development, such as achievements, for personalized education content, tailored learning paths, progress monitoring, and adaptive feedback within specific domains, such as pediatric diabetes self-management training. Adaptive learning models provide a theoretical basis for personalized goal setting and educational processes and analyze nursing records through natural language processing technology to identify weak links, thereby triggering targeted learning modules and enhancing learners' awareness of their personal learning progress.

Another research focus explores the application of adaptive learning in the field of education, particularly in medical education. Cluster #2 personalized learning supports tailored curricula for nurses at different career stages (e.g., new hires vs. seasoned staff). For instance, VR simulations could train novice nurses in high-risk scenarios [22]. Medical education and medical students, as a unique group within the educational field, have distinct learning requirements and approaches, and adaptive learning can provide more precise and efficient learning support for them [22, 23]. Mobile-based adaptive systems can deliver bite-sized training during shifts, and interactive learning environments facilitate team-based learning (e.g., virtual case discussion between nurses and physicians), which contribute to enhancing learning experiences and improving care coordination [23].

The evaluation of adaptive learning outcomes is also a research focus within this field. By analyzing learning data, collecting feedback from students or clinical nurses, and tracking application effects, the effectiveness of adaptive learning can be objectively assessed [24, 25]. This evaluation can be considered from aspects such as learning outcomes (degree of knowledge acquisition, skill enhancement, and learning pace), learning experience (satisfaction, engagement, and perception of personalization), and learning adaptability (adaptive adjustments, feedback mechanisms, and self-regulatory capabilities), and it supports the refinement and dissemination of adaptive learning technologies [26, 27]. Jevremovic et al. [28] created an adaptive learning tool that dynamically tailors learning paths based on student knowledge. It enhanced student performance in IP address tasks in computer networking, leading to more evenly distributed scores compared to nonusers. Chou et al. [29] developed an adaptive learning system incorporating help-seeking negotiation, promoting student autonomy and proactivity in assistance. Findings show that the experimental group excelled in independent problem-solving with less administrative help than the control group.

The burst keywords identified in our analysis reflect future research trajectories in adaptive learning research, including “outcomes,” “adaptive expertise,” “higher education,” “artificial intelligence,” “support,” “deep learning,” and “engagement.” Intelligent optimization and outcome-oriented adaptive learning are among the emerging trends in future research. The surge in AI-related research mirrors its transformative role in education, as highlighted by Darvishi et al. [11], who demonstrated AI's capacity to personalize learning paths through real-time data analytics. Our findings corroborate this, showing AI's dominance in recent publications, particularly in intelligent tutoring systems and personalized learning. The AI burst reflects adoption in higher education [30], while “outcomes” echoes Hooshyar et al.'s [25] emphasis on evidence-based evaluation. For example, Hooshyar et al. [25] conducted a meta-analysis to explore the efficacy of personalized TEL within higher education settings. The findings indicated that the implementation of personalized TEL significantly improves student learning outcomes, particularly in terms of cognitive achievement. Additionally, adaptive expertise resonates with Cutrer et al.'s [31] conceptual framework for “Master Adaptive Learners” in medical education, where adaptability is linked to long-term skill retention. Our cluster analysis further associates it with “performance” and “feedback,” underscoring its relevance to competency-based training in nursing. The burst of “adaptive expertise” also supports Mylopoulos et al.'s [32] call for curricula that foster adaptive skills.

### 4.1. Limitations and Future Research

Recognizing the limitations of our study is crucial. While the WoS database provides a broad multidisciplinary academic literature overview, our inclusion and exclusive criteria might have led to the omission of significant findings from alternative sources. Moreover, the data extraction methodology, guided by our literature review, could potentially introduce selection bias. To mitigate these limitations in subsequent studies, we intend to broaden our scope of data sources and refine our search techniques to enhance the robustness of our findings. Despite significant progress in adaptive learning technologies, critical gaps remain in nursing research. First, the applicability of these systems in resource-constrained settings, such as rural clinics or low-income regions, remains underexplored, which limits their scalability. Additionally, longitudinal data on skill retention and clinical competency decay following training are scarce, hindering the optimization of evidence-based programs. Third, most adaptive platforms lack alignment with standardized nursing competency frameworks, resulting in fragmented educational outcomes. Therefore, nursing administrators can integrate adaptive algorithms with a nationally recognized competency graph to automate personalized learning paths to ensure learning outcomes.

## 5. Conclusions

This investigation employed a bibliometric approach to assess the landscape of adaptive learning between 2014 and 2024, leveraging CiteSpace and data from the WoS. The study mapped the topical focal points and global trends in adaptive learning research, noting an increasing trend in publication volumes with the United States as the leading contributor. It also underscored the necessity for enhanced collaborative efforts across nations, institutions, and researchers. Key areas of interest within the field were pinpointed, including the theoretical and technological aspects of adaptive learning, its educational applications, and its impacts, indicating potential future research trajectories toward intelligent, outcome-based adaptive learning and its intersection with higher education.

### 5.1. Implications for Nursing Management

AI drives transformative changes in nursing management through adaptive learning technologies. Nursing administrators can leverage adaptive learning platforms such as VR simulations to identify knowledge gaps and deliver personalized training modules. For instance, AI algorithms can analyze nurses' performance in simulated scenarios to tailor follow-up exercises. Additionally, adaptive systems can predict staffing needs by analyzing historical patient data, enabling dynamic shift scheduling to maintain care quality during shortages.

## Figures and Tables

**Table 1:** Top 10 countries by number of publications.

Rank	Country	Publications, *n* (%)	Centrality
1	United States of America	214 (38.15)	0.64
2	China	58 (10.34)	0.15
3	Canada	45 (8.02)	0.12
4	Netherlands	32 (5.70)	0.05
5	Spain	29 (5.17)	0.01
6	Australia	26 (4.63)	0.08
7	England	24 (4.28)	0.09
8	Germany	22 (3.92)	0.01
9	India	16 (2.85)	0.00
10	Serbia	11 (1.96)	0.07

**Table 2:** Top 10 institutions by number of publications.

Rank	Institutions	Country	Publications, *n* (%)
1	University of Toronto	Canada	16 (2.85)
2	New York University	USA	13 (2.32)
3	Purdue University	USA	12 (2.14)
4	University of Michigan	USA	12 (2.14)
5	Arizona State University	USA	10 (1.78)
6	Stanford University	USA	10 (1.78)
7	Johns Hopkins University	USA	9 (1.60)
8	Harvard Medical School	USA	9 (1.60)
9	Beijing Normal University	China	9 (1.60)
10	American Medical Association	USA	8 (1.43)

**Table 3:** Top 10 authors who published adaptive learning research.

Rank	Author	Institutions	Publications, *n* (%)
1	Mylopoulos, Maria	University of Toronto, Canada	13 (2.32)
2	Woods, Nicole N	University of Toronto, Canada	7 (1.25)
3	Pusic, Martin, V	Harvard Medical School, USA	7 (1.25)
4	Bartholomew, Scott R.	Purdue University, USA	7 (1.25)
5	Cutrer, William B.	Vanderbilt University, USA	6 (1.07)
6	Gisondi, Michael A	Stanford University, USA	5 (0.89)
7	Branzetti, Jeremy	New York University, USA	5 (0.89)
8	Hopson, Laura R.	University of Michigan, USA	5 (0.89)
9	Regan, Linda	Johns Hopkins University, USA	5 (0.89)
10	Rau, Martina A	University of Wisconsin, USA	5 (0.89)

**Table 4:** Top 10 cited authors who published adaptive learning research.

Rank	Author	Publications, *n* (%)	Centrality
1	Brusilovsky P	42 (7.49)	0.13
2	Zimmerman BJ	41 (7.31)	0.24
3	Cohen J	40 (7.13)	0.08
4	Mayer RE	36 (6.42)	0.12
5	Cutrer WB	34 (6.06)	0.17
6	Mylopoulos M	31 (5.53)	0.03
7	Vanlehn K	30 (5.35)	0.09
8	Bandura A	30 (5.35)	0.13
9	Pintrich PR	29 (5.17)	0.10
10	Aleven V	29 (5.17)	0.03

**Table 5:** Top 10 cited journals in adaptive learning research.

Rank	Cited journal	Publications, *n* (%)	Impact factor (journal citation reports 2023)
1	*Computers and Education*	244 (43.49)	8.9
2	*Computers in Human Behavior*	157 (27.99)	9.0
3	*Medical Education*	135 (24.06)	4.9
4	*Academic Medicine*	129 (22.99)	5.3
5	*Review of Educational Research*	128 (22.82)	8.3
6	*Medical Teacher*	123 (21.93)	3.3
7	*Journal of Educational Psychology*	123 (21.93)	5.6
8	*Educational Psychologist*	122 (21.75)	14.3
9	*Educational Psychology Review*	110 (19.61)	10.1
10	*IEEE Transactions on Learning Technologies*	108 (19.25)	2.9

**Table 6:** Top 10 cited references by the adaptive learning research.

Rank	Title (author)	Year	Journal	Count	Centrality
1	Fostering the Development of Master Adaptive Learners: A Conceptual Model to Guide Skill Acquisition in Medical Education (Cutrer William B)	2017	*Academic Medicine*	21	0.04
2	Developing the Experts We Need: Fostering Adaptive Expertise Through Education (Mylopoulos Maria)	2018	*Journal of Evaluation in Clinical Practice*	16	0.03
3	A Scoping Review of Adaptive Expertise in Education (Kua Joanne)	2021	*Medical Teacher*	9	0.00
4	Twelve Tips for Designing Curricula That Support the Development of Adaptive Expertise (Mylopoulos Maria)	2015	*Medical Teacher*	9	0.01
5	Exploring the Characteristics and Context That Allow Master Adaptive Learners to Thrive (Cutrer William B)	2018	*Medical Teacher*	8	0.01
6	Adaptive Educational Hypermedia Accommodating Learning Styles: A Content Analysis of Publications From 2000 to 2011 (Akbulut Yavuz)	2012	*Computers and Education*	5	0.00
7	Using Adaptive Comparative Judgment for Student Formative Feedback and Learning During a Middle School Design Project (Bartholomew Scott R.)	2019	*International Journal of Technology and Design Education*	5	0.00
8	Active Learning Increases Student Performance in Science, Engineering, and Mathematics (Freeman S)	2014	*Proceedings of the National Academy of Sciences of the United States of America (PNAS)*	5	0.00
9	E-Learning Personalization Based on Hybrid Recommendation Strategy and Learning Style Identification (Klasnia-Milicevic, A)	2011	*Computers and Education*	5	0.00
10	Adaptive Distance Learning and Testing System (Markovic S)	2013	*Computers in Applied Engineering Education*	5	0.00

**Table 7:** Top 20 keywords that published papers on adaptive learning.

Rank	Keywords	Count	Centrality
1	Intelligent tutoring systems	80	0.11
2	Performance	64	0.14
3	Students	59	0.14
4	Education	53	0.18
5	Model	43	0.14
6	Medical education	43	0.05
7	Design	41	0.12
8	Knowledge	38	0.10
9	Impact	37	0.20
10	Task analysis	35	0.11
11	Strategy	30	0.10
12	Motivation	30	0.05
13	Science	29	0.06
14	Interactive learning environments	28	0.06
15	Teaching/learning strategies	28	0.10
16	Adaptive learning	27	0.06
17	Feedback	26	0.20
18	System	24	0.06
19	Adaptive expertise	24	0.01
20	Achievement	24	0.20

## Data Availability

The data that support the findings of this study are available from the corresponding author upon reasonable request.

## References

[1] Breda C. (2018). *Medical Knowledge Doubles Every Few Months; How Can Clinicians Keep Up?*.

[2] Cromer S. J., D’Silva K. M., Phadke N. A., Lord E., Rigotti N. A., Baer H. J. (2022). Gender Differences in the Amount and Type of Student Participation During In-Person and Virtual Classes in Academic Medicine Learning Environments. *JAMA Network Open*.

[3] Liu K., Zhang W., Li W., Wang T., Zheng Y. (2023). Effectiveness of Virtual Reality in Nursing Education: A Systematic Review and Meta-Analysis. *BMC Medical Education*.

[4] Chirikov I., Semenova T., Maloshonok N., Bettinger E., Kizilcec R. F. (2020). Online Education Platforms Scale College STEM Instruction With Equivalent Learning Outcomes at Lower Cost. *Science Advances*.

[5] Longhini J., Rossettini G., Palese A. (2021). Massive Open Online Courses for Nurses’ and Healthcare Professionals’ Continuous Education: A Scoping Review. *International Nursing Review*.

[6] Morris M. E., Brusco N. K., McAleer R. (2023). Professional Care Workforce: A Rapid Review of Evidence Supporting Methods of Recruitment, Retention, Safety, and Education. *Human Resources for Health*.

[7] Hahn-Schroeder H., Honig J., Smith C., Chin S., Frazier L. (2022). An Innovative Academic Practice Model for Clinical Nursing Education During the COVID-19 Pandemic. *Academic Medicine*.

[8] Kizilcec R. F., Pérez-Sanagustín M., Maldonado J. J. (2017). Self-Regulated Learning Strategies Predict Learner Behavior and Goal Attainment in Massive Open Online Courses. *Computers & Education*.

[9] Afini Normadhi N. B., Shuib L., Md Nasir H. N., Bimba A., Idris N., Balakrishnan V. (2019). Identification of Personal Traits in Adaptive Learning Environment: Systematic Literature Review. *Computers & Education*.

[10] Pliakos K., Joo S. H., Park J. Y., Cornillie F., Vens C., Van den Noortgate W. (2019). Integrating Machine Learning into Item Response Theory for Addressing the Cold Start Problem in Adaptive Learning Systems. *Computers & Education*.

[11] Darvishi A., Khosravi H., Sadiq S., Gašević D., Siemens G. (2024). Impact of AI Assistance on Student Agency. *Computers & Education*.

[12] Ipinnaiye O., Risquez A. (2024). Exploring Adaptive Learning, Learner-Content Interaction and Student Performance in Undergraduate Economics Classes. *Computers & Education*.

[13] Koskinen A., McMullen J., Hannula-Sormunen M., Ninaus M., Kiili K. (2023). The Strength and Direction of the Difficulty Adaptation Affect Situational Interest in Game-Based Learning. *Computers & Education*.

[14] Jamal N. N., Jawawi D., Hassan R. (2024). Adaptive Learning Framework for Learning Computational Thinking Using Educational Robotics. *Computer Applications in Engineering Education*.

[15] Wang J. J., Liang Y. Q., Cao S. M., Cai P. X., Fan Y. M. (2023). Application of Artificial Intelligence in Geriatric Care: Bibliometric Analysis. *Journal of Medical Internet Research*.

[16] Chen C. (2006). CiteSpace II: Detecting and Visualizing Emerging Trends and Transient Patterns in Scientific Literature. *Journal of the American Society for Information Science*.

[17] Chen C., Hu Z., Liu S., Tseng H. (2012). Emerging Trends in Regenerative Medicine: A Scientometric Analysis in CiteSpace. *Expert Opinion on Biological Therapy*.

[18] Hong Y. J., Saab N., Admiraal W. (2024). Approaches and Game Elements Used to Tailor Digital Gamification for Learning: A Systematic Literature Review. *Computers & Education*.

[19] Mao S., Zhan J. Y., Wang Y. Z., Jiang Y. C. (2023). Improving Knowledge Tracing via Considering Two Types of Actual Differences From Exercises and Prior Knowledge. *IEEE Transactions on Learning Technologies*.

[20] George G., Lal A. M. (2019). Review of Ontology-Based Recommender Systems in e-Learning. *Computers & Education*.

[21] Rijgersberg-Peters R., van Vught W., Broekens J., Neerincx M. A. (2024). Goal Ontology for Personalized Learning and Its Implementation in Child’s Health Self-Management Support. *IEEE Transactions on Learning Technologies*.

[22] Cutrer W. B., Spickard W. A., Triola M. M. (2021). Exploiting the Power of Information in Medical Education. *Medical Teacher*.

[23] Nathaniel T. I., Goodwin R. L., Fowler L., McPhail B., Black A. C. (2021). An Adaptive Blended Learning Model for the Implementation of an Integrated Medical Neuroscience Course During the COVID-19 Pandemic. *Anatomical Sciences Education*.

[24] Hillmayr D., Ziernwald L., Reinhold F., Hofer S., Reiss K. M. (2020). The Potential of Digital Tools to Enhance Mathematics and Science Learning in Secondary Schools: A Context-Specific Meta-Analysis. *Computers & Education*.

[25] Hooshyar D., Weng X. J., Sillat P. J., Tammets K., Wang M. H., Hämäläinen R. (2024). The Effectiveness of Personalized Technology-Enhanced Learning in Higher Education: A Meta-Analysis With Association Rule Mining. *Computers & Education*.

[26] Dinçer S., Doğanay A. (2017). The Effects of multiple-pedagogical Agents on Learners’ Academic Success, Motivation, and Cognitive Load. *Computers & Education*.

[27] Kok E. M., Niehorster D. C., van der Gijp A. (2024). The Effects of Gaze-Display Feedback on Medical Students’ Self-Monitoring and Learning in Radiology. *Advances in Health Sciences Education*.

[28] Jevremovic A., Shimic G., Veinovic M., Ristic N. (2017). IP Addressing: Problem-Based Learning Approach on Computer Networks. *IEEE Transactions on Learning Technologies*.

[29] Chou C. Y., Lai K. R., Chao P. Y., Tseng S. F., Liao T. Y. (2018). A Negotiation-Based Adaptive Learning System for Regulating Help-Seeking Behaviors. *Computers & Education*.

[30] Lu Y., Wang D. L., Chen P. H., Zhang Z. (2024). Design and Evaluation of Trustworthy Knowledge Tracing Model for Intelligent Tutoring System. *IEEE Transactions on Learning Technologies*.

[31] Cutrer W. B., Miller B., Pusic M. V. (2017). Fostering the Development of Master Adaptive Learners: A Conceptual Model to Guide Skill Acquisition in Medical Education. *Academic Medicine*.

[32] Mylopoulos M., Kulasegaram K., Woods N. N. (2018). Developing the Experts We Need: Fostering Adaptive Expertise Through Education. *Journal of Evaluation in Clinical Practice*.

